# The Role of Liquid Biopsy Analytes in Diagnosis, Treatment and Prognosis of Colorectal Cancer

**DOI:** 10.3389/fendo.2022.875442

**Published:** 2022-06-30

**Authors:** JinHua He, NaiTe Xi, ZePing Han, WenFeng Luo, Jian Shen, ShengBo Wang, JianHao Li, ZhongHui Guo, HanWei Cheng

**Affiliations:** ^1^ Central Laboratory of Panyu Central Hospital, Guangzhou Panyu Central Hospital, Guangzhou, China; ^2^ Department of Hepatobiliary Surgery, The First Affiliated Hospital of Jinan University, Guangzhou, China; ^3^ Department of Gastroenterology, Central Hospital of Panyu District, Guangzhou, China; ^4^ Institute of Cardiovascular Medicine, Central Hospital of Panyu District, Guangzhou, China

**Keywords:** colorectal cancer, circulating tumor cell, circulating tumor DNA, circulating tumor miRNA, exosomes

## Abstract

Colorectal cancer (CRC) is one of the most common malignant tumors of the digestive tract worldwide and is a serious threat to human life and health. CRC occurs and develops in a multi-step, multi-stage, and multi-gene process, in which abnormal gene expression plays an important role. CRC is currently diagnosed *via* endoscopy combined with tissue biopsy. Compared with tissue biopsy, liquid biopsy technology has received increasingly more attention and applications in the field of molecular detection due to its non-invasive, safe, comprehensive, and real-time dynamic nature. This review article discusses the application and limitations of current liquid biopsy analytes in the diagnosis, treatment, and prognosis of CRC, as well as directions for their future development.

## Introduction

Colorectal cancer (CRC) is one of the most common cancers worldwide. Due to changes in living environments, eating habits, and lifestyles, the incidence of CRC is increasing yearly in both developed and developing countries. There are approximately 1.8 million new cases worldwide each year, and approximately 900,000 people die from CRC every year (1, 2). Population growth and the aging of society may lead to a further increase in the number of deaths in many countries and regions, with the number of deaths from CRC predicted to double by 2035 (3). The long treatment process and lower quality of life of CRC not only impose a high financial burden on most patients’ families but also cause tremendous psychological pressure on patients (4).

To date, the diagnosis of CRC usually relies on the examination of serum biomarker levels, tissue biopsy, and imaging. However, the diagnostic accuracy and sensitivity of pathological and imaging methods are still limited, and the specificity of the commonly used serum markers is poor.

The main treatment for CRC is surgical resection. Pathological stage at the time of diagnosis can provide an accurate prediction for those who are at high risk of recurrence. Postoperative chemotherapy can be added to reduce this risk. Despite this, 17%–40% of CRC patients undergo relapse (5, 6). A study in the United States from 2001 to 2007 showed that the prognosis of patients diagnosed with local tumors, regional tumor spread, and distant tumor spread are significantly different with 5-year survival rates of 90.1%, 69.2%, and 11.7%, respectively (7). Tumor, node, metastasis (TNM) staging of the tumor is important and is also the basis for adjuvant chemotherapy. Therefore, clinical scientists have been looking for reliable biomarkers to provide guidance for the early diagnosis, treatment, and prognosis of CRC.

Traditional serum tumor markers such as carcinoembryonic antigen, carbohydrate antigen 19-9, and carbohydrate antigen 72-4 have, to a certain extent, guiding significance for CRC. Studies have shown that the combined quantification of these three markers is more sensitive than individually, but specificity is reduced; compared with patients with normal levels of these markers, the overall survival time of patients with elevated levels of all three markers is significantly shorter and the recurrence rate is higher (8).

In the past 30 years, some progress has been made in the use of genetic biomarkers such as plasmacytoma variant translocation 1, long-chain non-coding RNAs, and *KRAS* gene. The levels of these markers may be increased in patients with CRC and they have certain guiding significance for diagnosis (9, 10). However, traditional serum tumor markers or gene-level biomarkers are not sufficiently specific or sensitive, and there are large individual differences in their levels. Currently, there is a lack of biomarkers that can guide early diagnosis, targeted therapy, prognosis, and monitoring of patients with CRC.

Liquid biopsy technology, as a branch of *in vitro* diagnosis, refers to a non-invasive blood test that can be used to monitor circulating tumor cells (CTCs), circulating tumor DNA (ctDNA) fragments, metabolites, and so on released into the blood by tumors or metastases. It is a breakthrough technology for the use of adjuvant treatment in cancer. Its main advantages are as follows: it can address the problems associated with precision medicine; it reduces harm from biopsies through the use of non-invasive sampling, effectively prolonging the survival period of patients; and (iii) it is cost effective (11–16). Liquid biopsy analytes include mainly CTCs, ctDNA, circulating tumor RNAs, circulating tumor microRNAs (ctmiRNAs), circulating free DNA (cfDNA), exosomes, extracellular vesicles, and metabolites. In this review, we discuss the role of CTCs, ctDNA, exosomes, ctmiRNAs, and metabolites and outline their use as potential biomarkers in the diagnosis, treatment, and prognosis of CRC.

## CTCs

**Figure 1:**
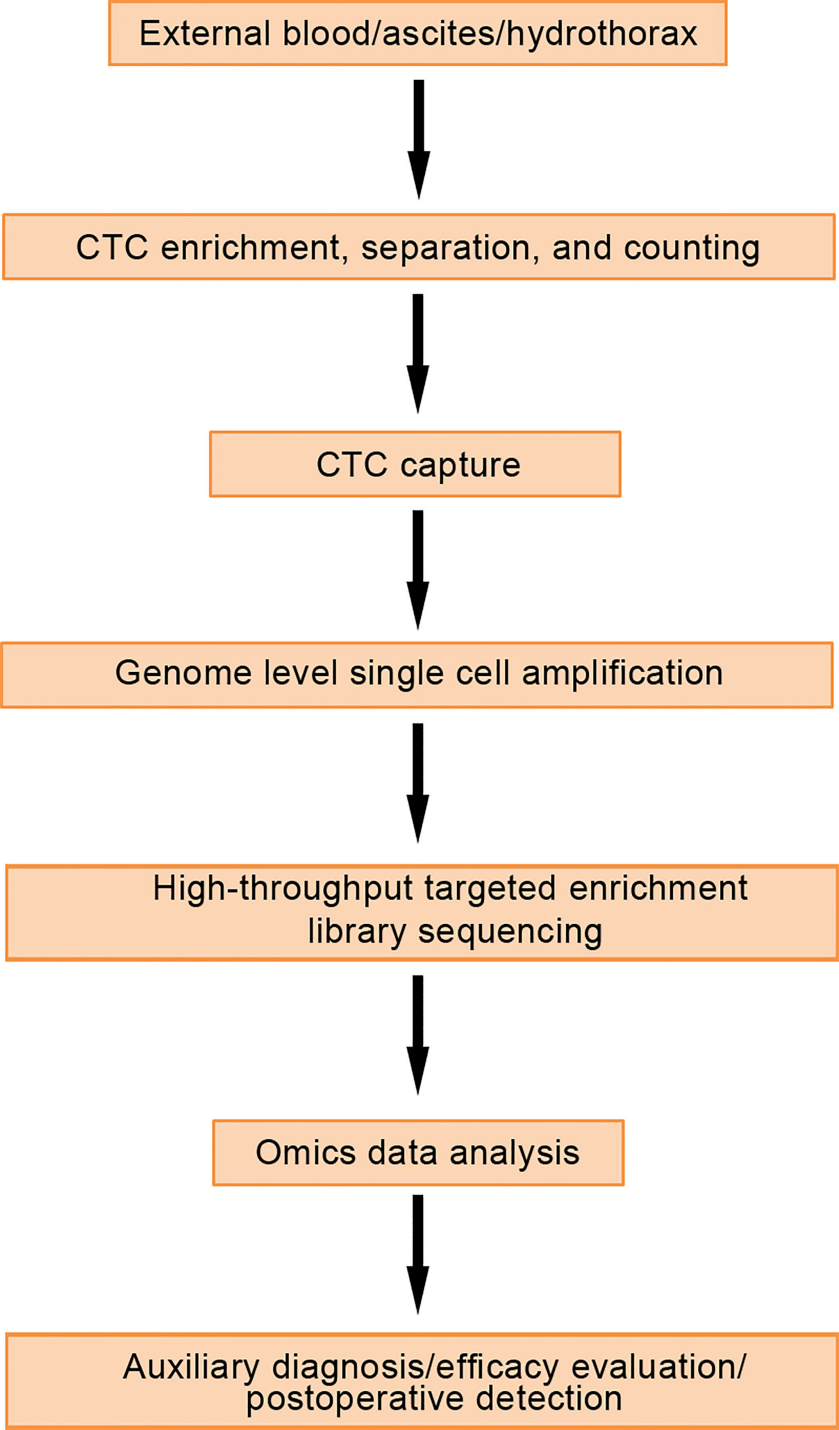
Detection process and application of circulating tumor cells.

CTCs are tumor cells that separate from tumor tissue and enter the circulatory system. The production of CTCs is a necessary condition for patients to develop a distant metastasis (17). CTCs detection technology mainly includes CTCs separation and enrichment technology and CTCs identification technology. The ideal separation, enrichment and detection technique of CTCs should meet the requirements of high sensitivity and specificity. CTCs must not be contaminated, and the collected CTCs can be identified and analyzed genetically (**Figure 1**). CTCs are not only an important prognostic indicator, they are also considered a very effective real-time biopsy tool and a window into the molecular mechanisms for pathogenicity and drug resistance in tumor cells (18). However, most CTCs die from shear stress, oxidative stress, and attack from the immune system. In the early stage of CRC, due to limitations of the detection methods, very few CTCs can be captured in the peripheral blood of CRC patients, and it is almost impossible to identify CTCs in the blood of early CRC patients, which limits the possibility of using CTCs for clinical analysis. After decades of development, tremendous breakthroughs have been made in the technology for detecting CTCs, including nucleic acid identification based on PCR, and enrichment methods using the physical and biological characteristics of CTCs (19, 20). Recently, Cheng et al. developed Hydro-Seq, a new type of microfluidic chip used to capture CTCs that can accurately separate ultra-high purity CTCs from patient blood samples, independent of white blood cells and red blood cells. By using Hydro-Seq, comprehensive analysis of CTCs can be performed in a high-throughput manner, which can effectively provide patients with treatment programs in clinical practice (21).

Based on the progress of CTC detection methods, researchers have performed in-depth studies on CTCs in CRC. In terms of early screening, in 620 patients (including patients with stage I–IV CRC and precancerous lesions), the quantitative detection of CTCs is generally accurate in judging the stage of all colorectal diseases including precancerous lesions with an accuracy of 88% (22). In terms of the clinical stage of the disease, the number of CTCs reflects the degree of tumor mutation in patients, to a certain extent. In patients with advanced CRC, the detection rate of CTCs increases as the tumor stage increases. Further research on the TNM staging system has shown that the number of detectable CTCs is positively correlated with the size and depth of invasion of the primary tumor, lymph node invasion, and distant metastasis, suggesting that CTCs can be used to judge lymph node invasion and distant metastasis (23–26).

In a study of 183 CRC patients, blood samples were collected at different time points during the pre-follow-up and follow-up periods. The results showed that the presence of CTCs before surgery was associated with a significant reduction in survival, and patients with a high risk of recurrence could be identified (27). Other studies have also shown that the presence of CTCs is an indicator for the poor prognosis of CRC patients (28, 29). The total number of CTCs and the number of CTCs with a mesenchymal phenotype are reported to be significantly related to advanced disease stage and the occurrence of metastasis (30). The total number of mesenchymal-type CTCs can be used as a prognostic marker of disease progression and metastasis in CRC patients. For example, 149 CRC patients were tested for CTCs according to tumor stage (TNM); as tumor stage increased, the number of CTCs also increased. Survival analysis showed that with the increase in T stage, the number of CTCs increased and the survival and risk curves changed faster (31). In addition, Nicolazzo et al. compared the characteristics of CTCs in the blood of 84 metastatic colon cancer patients with left colon cancer (LCC) and right colon cancer (RCC) and found that the CTCs present in both types of colon cancer showed phenotypic heterogeneity (20). Compared with proximal primary tumors, the CTCs found in patients with distal primary tumors are mainly mesenchymal. The CTCs of RCC patients are mostly apoptotic, whereas those of LCC patients are mainly mesenchymal. This may indicate that the biology of proximal and distal cancers differs substantially, which might be related to the different modes of tumor cell spread. The poor prognosis of RCC is not determined by the hematological dissemination of tumor cells, which is manifested mainly by the passive shedding of inactive cells. In contrast, subtypes of LCC with a poor prognosis can be identified reliably by the presence of mesenchyme-type CTCs (18).

## cfDNA and ctDNA

**Figure 2:**
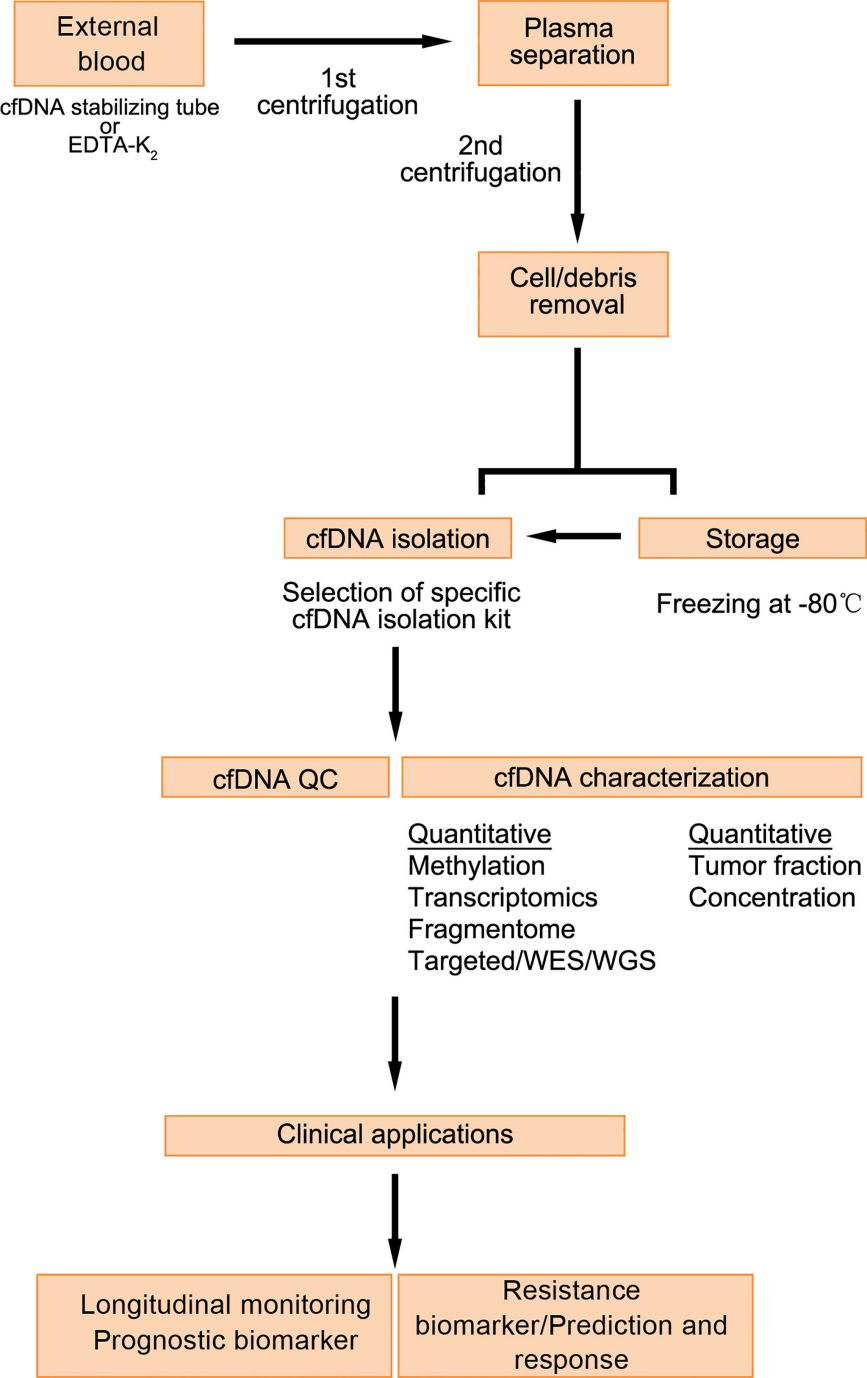
Detection process and application of cfDNA (33).

cfDNA usually refers to degraded DNA fragments that are released into the plasma. cfDNA exists in various human body fluids. Under physiological conditions, cfDNA in the blood comes mainly from the necrosis and apoptosis of white blood cells. In some diseases and under special conditions, other cells also release cfDNA into the blood, including ctDNA derived from tumor cells. ctDNA fragments are derived mainly from necrotic tumor cells, apoptotic tumor cells, circulating tumor cells, and exosomes secreted by tumor cells (32). At present,cfDNA detection methods mainly include real-time fluorescence quantitative PCR technology, digital PCR (dPCR) technology, high-throughput sequencing (NGS) technology. Flight mass spectrometry. Detecting changes in the number of types of cfDNA in specific sequences can be used to identify cancer, and related diseases. Measuring the size of cfDNA can reveal which cell death mechanism it is produced by (**Figure 2**).

Under normal circumstances, cfDNA is produced mainly as small and uniform 185-200-bp fragments during cell apoptosis. Tumor cells undergo unique biological processes, including necrosis, apoptosis, and extracellular transport, resulting in large cfDNA fragments of different sizes greater than 200 bp (34). Interestingly, the proportion of cfDNA fragments smaller than 100 bp is higher in CRC patients than in healthy individuals, and the proportion of cfDNA fragments larger than 300 bp is lower in CRC patients than in healthy individuals (35). Although the consistency between cfDNA and genomic DNA in tumor biopsies is still controversial, there is evidence that there is a high degree of consistency between the two (34).Kang et al. performed ultra-deep sequencing of 10 genes (38 kb) with repeated mutations from plasma cfDNA, peripheral blood mononuclear cell genomic DNA, and available genomic DNA from matching tumor tissues from 54 patients with metastatic CRC. Analysis showed that the two types of samples generally had a high degree of coincidence, with a coincidence rate of 93% (34). Studies have pointed out that the level of cfDNA is significantly increased in the blood of patients with metastatic CRC (35). In comparison with other colorectal-related diseases, the cfDNA level of patients with primary CRC is significantly higher than that of patients with intestinal polyps (36).

As common CRC mutation genes, *KRAS* and *BRAF* have a high detection value. cfDNA analysis can be used to detect KRAS and BRAF mutations. In the case of KRAS mutations, cfDNA analysis has diagnostic value for patients with a poor prognosis (37, 38). In addition, analysis of *BRAF* mutations in cfDNA can be used as a prognostic indicator for CRC. In patients with high levels of *BRAF* mutations, overall survival is usually low (39). Similarly, the detection of *BRAF* mutations in cfDNA plays an important role in the selection of treatment options for CRC patients (40). Siravegna et al. used digital PCR to quantitatively analyze the mutation levels of undetermined genes in the ctDNA of metastatic CRC patients, revealing the dynamic process of tumor evolution in the process of drug therapy, and also providing a basis for metastatic CRC-targeted therapy strategies (41). These studies have shown that the detection of cfDNA has guiding significance for the diagnosis, prognosis, and postoperative chemotherapy of CRC patients.

Because genetic and epigenetic changes affect the development of CRC, the use of DNA methylation as a biomarker to improve current diagnosis, screening, prognosis, and treatment has great potential (42). Abnormal methylation patterns can be detected in cfDNA. For example, the hypermethylation of *ALX4, FBN2, HLTF, P16, TMEFF1, and VIM* in cfDNA is associated with poor prognosis in patients with CRC. At the same time, the hypermethylation of *APC, NEUROG1, RASSF1A, RASSF2A, SDC2, SEPT9, TAC1, and THBD* in cfDNA can be detected in patients with early CRC, while the hypermethylation of *P16 and TFPI2* in cfDNA is related to the recurrence of CRC (43). The methylation status of the *SEPT9* gene promoter in cfDNA can be used as a biomarker for detecting early CRC with a sensitivity of 30%–75% and specificity of approximately 90%. The large difference in sensitivity and specificity may be related to the relatively small number of patients examined and the technology used to detect SEPT9 methylation (44–48).

## ctmiRNAs

**Figure 3:**
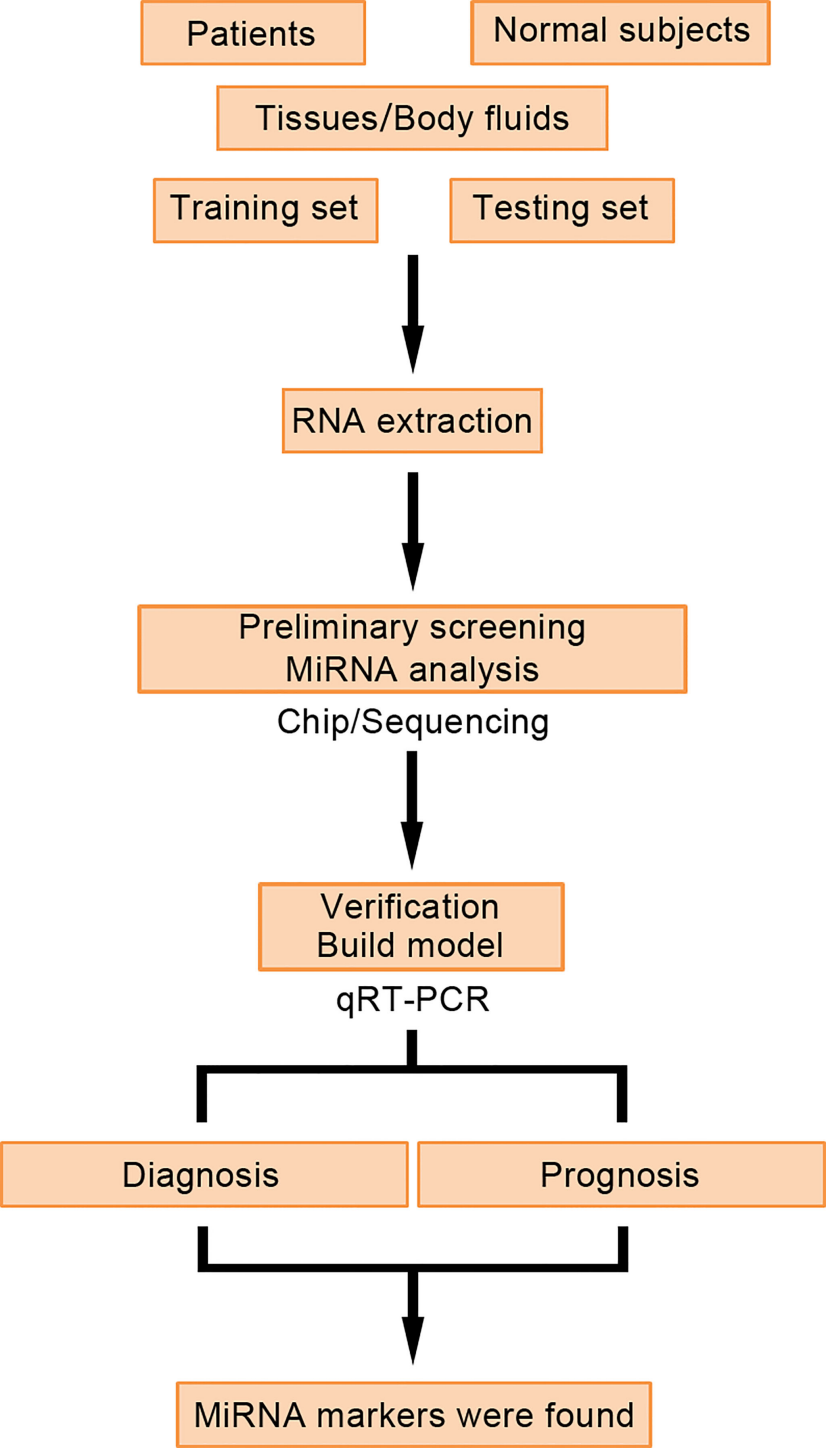
Detection process and application of ctmiRNAs.

miRNAs are a group of non-coding RNAs, 18–25 nucleotides in length, whose main function is to affect the expression of post-translational genes. They work by binding to the 3′-untranslated region of their target gene mRNA (49–51). The expression of miRNAs is related to tumor tissue type, degree of differentiation, aggressiveness, treatment prognosis, and other clinical biological characteristics (52). Tumor cells release miRNAs specific to their metabolism into the peripheral blood, which are called ctmiRNAs, and they are carried by exosomes, which can protect them from RNase degradation (53, 54). The detection of ctmiRNAs was verified by fluorescence probe q-PCR. Specific miRNA primers and joint primers were used for rapid amplification of miRNA during the detection process. At the same time, fluorescence probe was used to combine the detection process to ensure high specificity and sensitivity of the amplification process. miRNA molecules can be specifically detected and miRNA molecules with high similarity can be distinguished (**Figure 3**). Therefore, the detection of ctmiRNAs in the serum or plasma of CRC patients has great potential for diagnosis, prognosis, and treatment guidance.

ctmiRNAs can be used not only to screen patients with CRC but also to distinguish CRC from other gastrointestinal tumors. As early as 2008, Hunter et al. found that a subset of 69 ctmiRNAs were highly expressed in the serum of CRC patients but were almost undetectable in the serum of healthy volunteers (55) Kosaka et al. performed ctmiRNA expression profile analysis using tissue and plasma samples from CRC patients and healthy volunteers. They found that the levels of *miR-17-3p* and *miR-92* were significantly increased in the serum of CRC patients; however, after surgical removal of the tumor, their expression was significantly decreased, suggesting that the expression of *miR-17-3p and miR-92* in serum was specific for screening CRC (56). *miR-21* is a secreted miRNA and one of the most intensively studied ctmiRNAs. It is usually released by cancer cells and is abundant in plasma and serum (57). miR-21 participates in the metabolic process of CRC by targeting the expression of *PTEN, PDCD, and DKK2* and activating the *Wnt/β*-catenin signaling pathway (58–60). Some studies have pointed out that the expression level of miR-21 in the serum of CRC patients is positively correlated with its expression level in tumor tissues, and miR-21 expression in the serum is significantly higher in CRC patients than in patients with colorectal adenoma and healthy individuals. Therefore, serum miR-21 may become a promising biomarker for screening CRC patients (61–64).

## Exosomes

**Figure 4:**
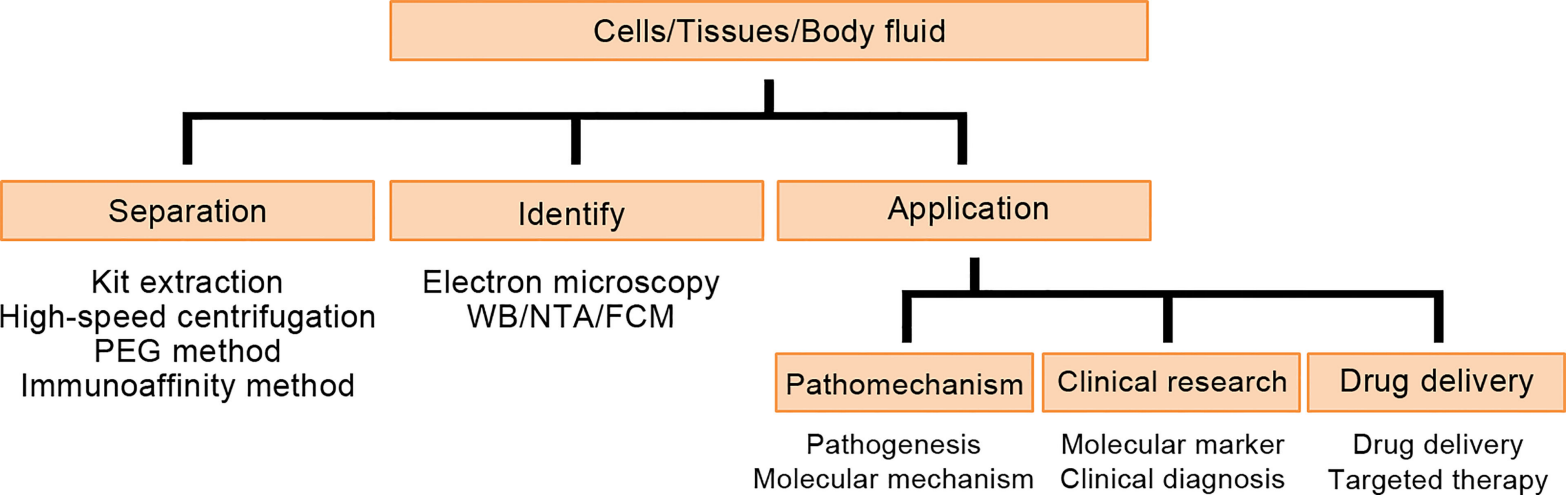
Detection process and application of exosomes.

Exosomes are small vesicles secreted by cells that contain proteins, DNA, mRNAs, and some non-coding RNAs (65). At present, exosomes are mostly isolated by ultrafast centrifugation, magnetic bead immunocapture, precipitation or filtration. The size and morphology were analyzed by electron microscopy, cell surface markers were analyzed by flow cytometry, proteins were analyzed by Western blot and ELISA, or RNA was analyzed by qPCR and next-generation sequencing (**Figure 4**).

They are means of communication between cells. Studies have found that exosomes are related to the occurrence and development of tumors, and there is a correlation between metastasis and drug resistance (66–68). Tumor cells use exosomes as carriers to help them escape immune system surveillance. On the one hand, exosomes guide the direction of tumor cell metastasis, and on the other hand, they also create a microenvironment suitable for tumor growth. The information carried by exosomes is diverse (69, 70). The proteins and nucleic acids they carry can be used for the early diagnosis of cancer, recurrence monitoring, drug resistance monitoring, and other related analyses (71, 72). Moreover, exosomes are more abundant than CTCs, which makes them easier to be enriched. Exosomes effectively protect the nucleic acids they carry from degradation, which overcomes the problem that ctDNA is easily degraded in blood, and have broad prospects in clinical applications.

Exosomes are involved in the occurrence and development of a variety of malignant tumors and have been considered by many scholars as a reliable biomarker that can be used to assist cancer prediction and diagnosis and guide treatment (73, 74)Exosomes involved in the occurrence and progression of malignant tumors are called tumor-derived exosomes (TDEs). TDEs are widely involved in tumor cell proliferation and metastasis and mediate the transmission of information between tumor cells and the tumor microenvironment, thereby regulating related signaling pathways, transformation of recipient cells, and expression of tumor genes. The tumor-associated miRNAs carried by TDEs interact with stromal cells in the tumor microenvironment to regulate tumor progression, angiogenesis, and immune escape, thereby causing changes in cell metabolism (75). In a recent study, Shang et al. identified a new type of CRC-derived exosomal circular RNA, called *circPACRGL*, which is a circulating exosomal RNA secreted by CRC cells (76) *circPACRGL* acts as a “sponge” to adsorb *miR-142-3p/miR-506-3p* and promote the proliferation, migration, and invasion of CRC cells through the transforming growth factor-β1 axis (75). However, TDEs do not only affect the gene expression of malignant tumors, they also participate in the distant metastasis of malignant tumors. For example, CXCL13 induces the formation of a pre-metastatic niche, which in turn promotes liver metastasis of CRC (76). Zhao et al. found that CRC cell-derived exosomes mediate miR-934 to induce the polarization of M2 macrophages by down-regulating the expression of PTEN and activating the PI3K/AKT signaling pathway. Polarized M2 macrophages can secrete chemokines (77). Liu et al. found that exosome-mediated miR-106b-3p promotes distant metastasis of CRC by down-regulating the expression of *DLC-1* (78). Zou et al. reported that serum exosome-mediated *miR-150-5p* expression levels are significantly reduced in CRC patients and closely related to poor differentiation, positive lymph node metastasis, and advanced TNM staging (79). Similarly, Liu et al. found that circulating exosome-mediated *miR-27a/miR-130*a are highly expressed in the plasma of CRC patients, and their plasma concentration is negatively correlated with prognosis (80). Ren et al. found that miR-196b-5p is highly enriched in serum exosomes of CRC patients and related to poor prognosis (81). Some exosome-mediated miRNAs can also be used to predict treatment response, for example, *miR-21-5p, miR-1246, miR-1229-5p, and miR-96-5p* in 5-FU/oxaliplatin therapy. Their expression is significantly higher in oxaliplatin-resistant patients than in a sensitive control group, which shows that they have great value in identifying 5-FU/oxaliplatin-resistant/sensitive patients (82). Exosome-mediated miRNAs can also be used as biomarkers for recurrence in CRC patients. For example, the expression level of *miR-17-92a* mediated by exosomes in serum is related to the recurrence of CRC, and patients with high serum *miR-17-92a* levels often have a poor prognosis (83)

Although there have been a large number of studies, research assessing the feasibility of exosomes as biomarkers in malignant tumors is only in its infancy. The main disadvantage of using exosomes is the lack of a technical consensus, which leads to differences in the results presented by different technologies. Therefore, before exosomes become clinical biomarkers, efforts must be made to standardize every procedure for their detection.

## Metabolomics

**Figure 5:**
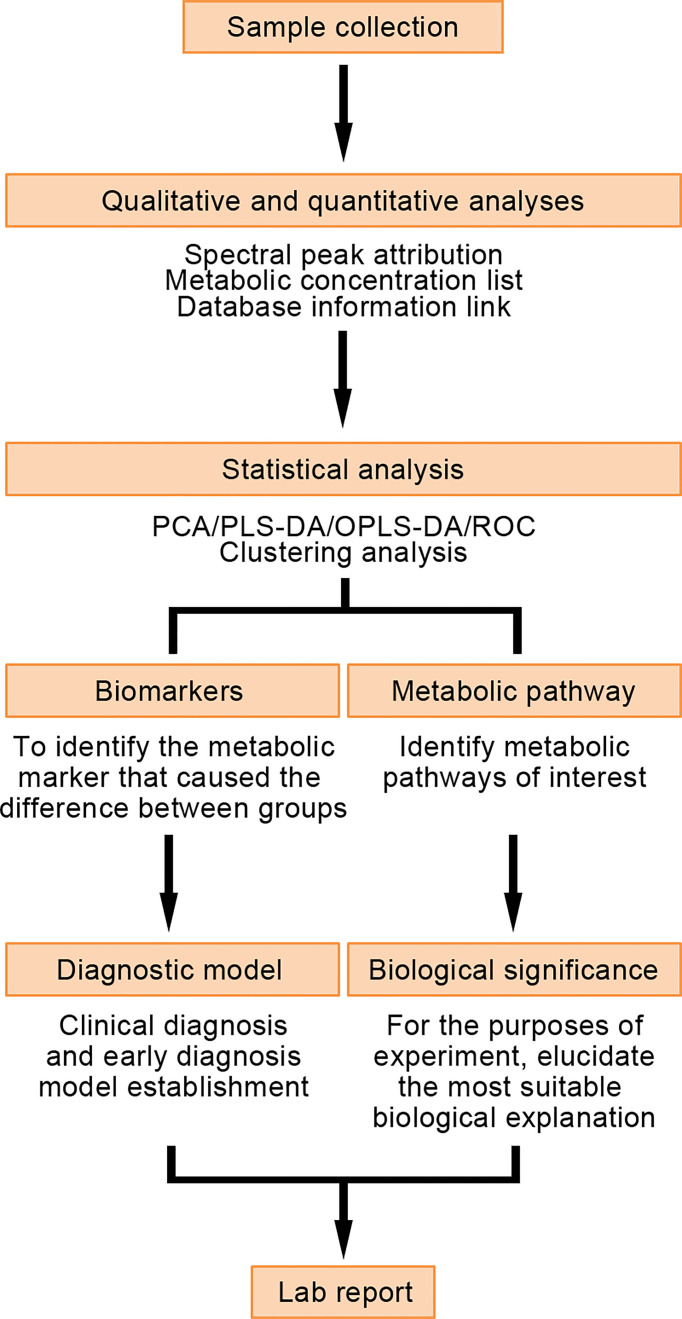
Detection process and application of metabolomics.

Metabolomics is an important branch of systems biology as well as a supplement to genomics, transcriptomics, and proteomics. Metabolomics integrates information modeling and systems mainly through high-throughput detection and data processing (84). Metabolomics can identify metabolites with a molecular weight of less than 1 kDa, such as sugars, lipids, and vitamins. The changes in their metabolic levels can be used to determine pathophysiological state at a certain period of time. Small molecular metabolites in this state can become markers for disease screening and early diagnosis (85). The research process for metabolomics involves sample preparation, detection and analysis of the metabolites in the sample, and statistical processing of the data obtained from metabolite analysis of the sample (**Figure 5**). Samples for detection and analysis are often biological fluids (urine and blood), cells, or tissues that can be obtained easily in a non-invasive manner (86).

Serum has always been the sample of choice for the identification of metabolites because it is easy to obtain and reflects a patient’s metabolic profile at the time of collection. Qiu et al. analyzed the serum metabolomics characteristics of 64 CRC patients and 65 healthy individuals using two methods, i.e., gas chromatography high-throughput time-of-flight (TOF) mass spectrometry (MS) and ultra-high pressure liquid chromatography quadruple-TOF-MS, and identified 33 different metabolites, among which the abnormal metabolism of glycolysis, arginine, proline, and fatty acid oleamide may be related to the pathogenesis of CRC (87). Uchiyama et al. used capillary electrophoresis-TOF-MS to analyze samples from 56 patients with CRC, 59 patients with colon adenoma, and 60 healthy individuals, and identified 139 metabolites. Among them, benzoic acid may be a diagnostic marker for CRC (88).

The analysis of metabolites in plasma is not only limited to CRC and healthy individuals but can also be used for precancerous lesions related to CRC. Gumpenberger et al. compared the plasma metabolites of 88 patients with CRC and 400 patients with colorectal adenoma. Compared with colorectal adenoma patients, the plasma levels of 1-methylnicotinamide and carnitine were higher, the concentrations of bilirubin, lysolecithin, choline, and polyunsaturated fatty acids were decreased, while taurine, caffeine-related metabolites, and hypoxanthine were increased in patients with CRC (89).

In patients with CRC at different stages, the analysis of metabolites in plasma also has a certain guiding role. For example, in serum metabolite analysis of 744 patients with stage I–IV CRC, compared with stage I patients, there was no significant difference in plasma metabolite concentrations in stage II patients; sphingomyelin C26:0 concentration was significantly reduced and sphingomyelin C18:0 and phosphatidylcholine C32:0 plasma concentrations were increased in stage III patients; citrulline, histidine, phosphatidylcholine (diacyl) C34:4, and phospholipids were significantly reduced and acylcholine (acyl-alkyl) C40:1 and lysophosphatidylcholine (acyl) C16:0 and C17:0 were significantly reduced in stage IV patients (90).

In addition, metabolite analysis has guiding significance for the differentiation of LCC and RCC. Deng et al. used ultra-high performance liquid chromatography quadruple-TOF-MS to analyze the plasma metabolites of 147 patients with LCC and 105 patients with RCC. Compared with LCC patients, anserine, l-arginine, gamma-glutamyl gamma-aminobutyraldehyde, and pyridoxal 5′-phosphate levels were reduced in RCC patients. In contrast, compared with LCC patients, trimethylamine oxide and indoxyl sulfate levels were increased in RCC patients (91).

## Conclusions

In summary, liquid biopsies have great significance for the individualized and precise treatment of CRC. For liquid biopsy analytes, ctDNA, CTCs, ctmiRNAs, exosomes, and metabolites have their own advantages and disadvantages (**Table 1**). In essence, they all obtain tumor genetic material and metabolites for molecular diagnosis. They should not exclude or oppose each other but should be complementary, reflecting the characteristics of tumors from different times, areas, and perspectives. Obviously, the more accurate and comprehensive our understanding of the molecular characteristics of tumors more targeted and precise treatment strategies can be developed. However, the constantly changing characteristics of tumor heterogeneity and the fact that tissue specimens are not easy to obtain have made the role and value of liquid biopsies increasingly important. We believe that with the continued improvement and development of liquid biopsy-related technologies, liquid biopsies will play an increasingly important role in individualized precision medicine for CRC.

**Table 1:** Comparison of advantages and disadvantages of liquid biopsy technology.

	Advantages	Disadvantages	Applications
CTCs	Tumor source specificity. Contain a huge amount of information, including but not limited to DNA, RNA, and proteins. Live cell culture can be carried out to facilitate subsequent functional research. The cells are easy to culture and expand, and a large number of target cells can be obtained.	Enrichment technology is not mature, their concentration in circulating blood is low, and capture technology is poorly developed. Specificity is poor: individual patients are usually different, and different tumor stages and types and drug effects affect CTCs to a certain extent. There are similar differences in different disease periods in the same patient.	Analyze prognosis. Assess the effect of treatment. Detect recurrence.
ctDNA/cfDNA	Can fully reflect the mutation information of the tumor at the DNA level. ctDNA is derived from the specific mutation of malignant tumor cells and has a high degree of specificity, which can provide guidance for targeted drug therapy.	The early detection rate of cancer is relatively low, while that of advanced cancer is relatively high. The source is complex, individual differences between different patients are huge, there are many gene mutations in different patients, and it is difficult to establish a unified detection standard. Do not reflect mutations at the non-DNA level, such as post-translational modifications of proteins. Difficult to determine the source.	Individualized medication. Assess the effect of treatment. Early auxiliary diagnosis.
Exosomes	High levels in blood and easy to enrich. High stability, can exist in blood for a long time, and can be used for *in vitro* research. Contain a large amount of information, including DNA, RNA, and protein, of malignant tumors. Wide applicability and great potential in early cancer screening.	The research started late and currently consists mostly of small sample research; there is a lack of large amount of experimental and clinical data support. Purification technology for exosomes is not yet mature.	Early screening for cancer.
ctmiRNAs	High levels in blood, easy to enrich, and the detection technology is mature. High specificity and sensitivity. Tumors larger than 0.2 cm can be detected, which can be used as a marker for early screening.	The research started late and there is no unified testing standard. The lack of large sample clinical data makes it impossible to determine whether their application in clinical practice is effective.	Early screening for cancer.
Metabolomics	Comprehensive, multi-level, and systematic analysis of a series of related metabolites with convincing results. There are fewer types of metabolites, the molecular structures of the substances are simple, and the analysis is simple and clear. Small changes in gene and protein expression will be amplified at the metabolite level, making detection easier.	There is no complete set of standard datasets that can be used in research and clinical practice. Metabolomics as systematic evidence has not yet established data integration with other related systems.	Disease screening. Early diagnosis.

## Author Contributions

JHH, NTX and ZPH wrote the original draft, JS, SBW, ZHG and WFL reviewed and edited the manuscript. HWC and JHL supervised the writing. All authors have contributed the manuscript and approved the submitted version.

## Funding

This work was supported by Science and Technology Project of Panyu District, Guangzhou (2019-Z04-02; 2020-Z04-026; 2021-Z04-053), Guangzhou Health and Family Planning Commission Program (No. 20181A011118; 20192A011027; 20191A011119; 20201A010085; 20212A010025), Guangzhou Science and Technology Plan Project (No. 202002030032 ), Medical Science and Technology Research Foundation of Guangdong Province (No. A2020304; A2022524), Scientific Research Fund project of Guangzhou Panyu Central Hospital (No. 2021Y002; 2021Y004).

## Conflict of Interest

The authors declare that the research was conducted in the absence of any commercial or financial relationships that could be construed as a potential conflict of interest.

## Publisher’s Note

All claims expressed in this article are solely those of the authors and do not necessarily represent those of their affiliated organizations, or those of the publisher, the editors and the reviewers. Any product that may be evaluated in this article, or claim that may be made by its manufacturer, is not guaranteed or endorsed by the publisher.

## Abbreviations

CRC, Colorectal Cancer; TNM, T, Primary Tumor; N, Regional Lymph Nodes, M: Distant Metastasis; CTC, Circulating Tumor Cell; CtDNA, Circulating Tumor DNA; CtmiRNA, Circulating Tumor miRNA; CfDNA, Circulating Free DNA; LCC, Left colon cancer; RCC, Right colon cancer; PBMC, Peripheral blood mononuclear cell; OS, Overall survival; PCR, Polymerase Chain Reaction; mCRC, Metastatic colon cancer; ALX4, Aristaless-like Homeobox 4; FBN2, fibrillin-2; HLTF, Helicase-like transcription factor; TMEFF1, transmembrane protein with EGF-like and two follistatinlike domains 1; VIM, vimentin; APC, Adenomatous Polyposis Colon; NEUROG1, Neurogenin 1; P16, Cyclin-dependent kinase inhibitor 2a; RASSF1A, Ras association domain family member 1; RASSF2A, Ras association domain family member 2; SDC2, Syndecan 2; SEPT9, Septin 9; TAC1, tachykinin precursor 1; THBD, Thrombomodulin; TFPI, Tissue factor pathway inhibitor 2; CRCR, Colorectal cancer recurrence; PTEN, phosphatase and tensin homolog deleted on chromosome ten; PDCD, Programmed cell death; DKK2, Dickkopf 2; CRA, colorectal adenoma; TDE, Tumor-derived exosomes; M2, Macrophages 2; CXCL13, chemokine (C-X-C motif) ligand 13; DLC-1, Deleted in Liver Cancer-1; GC-TOF-MS, Gas Chromatography High Throughput Time of Flight Mass Spectrometry; UPLC-QTOF-MS, Ultra high pressure liquid chromatography time-of-flight mass spectrometer; LysoPC, Lysophosphatidylcholine; EV, Extracellular vesicle foam; ELISA, Enzyme-Linked Immunosorbnent Assay.
